# Use of 21-Valent Pneumococcal Conjugate Vaccine Among U.S. Adults: Recommendations of the Advisory Committee on Immunization Practices — United States, 2024

**DOI:** 10.15585/mmwr.mm7336a3

**Published:** 2024-09-12

**Authors:** Miwako Kobayashi, Andrew J. Leidner, Ryan Gierke, Jennifer L. Farrar, Rebecca L. Morgan, Doug Campos-Outcalt, Robert Schechter, Katherine A. Poehling, Sarah S. Long, Jamie Loehr, Adam L. Cohen

**Affiliations:** ^1^Division of Bacterial Diseases, National Center for Immunization and Respiratory Diseases, CDC; ^2^Immunization Services Division, National Center for Immunization and Respiratory Diseases, CDC; ^3^Department of Health Research Methods, Evidence, and Impact, McMaster University, Hamilton, Ontario, Canada; ^4^College of Medicine and Public Health, University of Arizona, Phoenix, Arizona; ^5^California Department of Public Health; ^6^Wake Forest School of Medicine, Winston-Salem, North Carolina; ^7^Drexel University College of Medicine, Philadelphia, Pennsylvania; ^8^Cayuga Family Medicine, Ithaca, New York.

**Summary**

**What is already known about this topic?**

Adults aged 19–64 years with risk conditions for pneumococcal disease and those aged ≥65 years are recommended to receive either 15- or 20-valent pneumococcal conjugate vaccine (PCV) (PCV15 or PCV20, respectively).

**What is added by this report?**

On June 27, 2024, the Advisory Committee on Immunization Practices recommended 21-valent PCV (PCV21) as an option for adults aged ≥19 years who are currently recommended to receive PCV15 or PCV20. PCV21 contains eight serotypes not included in other licensed vaccines.

**What are the implications for public health practice?**

Adding PCV21 as an option in the current PCV recommendation is expected to prevent additional disease caused by pneumococcal serotypes unique to PCV21. Postlicensure monitoring of safety and public health impact of PCV use will guide future recommendations.

## Abstract

On June 17, 2024, the Food and Drug Administration approved 21-valent pneumococcal conjugate vaccine (PCV) (PCV21; CAPVAXIVE; Merck Sharp & Dohme, LLC) for adults aged ≥18 years. PCV21 does not contain certain serotypes that are included in other licensed pneumococcal vaccines but adds eight new serotypes. The Advisory Committee on Immunization Practices (ACIP) recommends use of a PCV for all adults aged ≥65 years, as well as adults aged 19–64 years with certain risk conditions for pneumococcal disease if they have not received a PCV or whose vaccination history is unknown. Previously, options included either 20-valent PCV (PCV20; Prevnar20; Wyeth Pharmaceuticals, Inc.) alone or a 15-valent PCV (PCV15; VAXNEUVANCE; Merck Sharp & Dohme, LLC) in series with 23-valent pneumococcal polysaccharide vaccine (PPSV23; Pneumovax23; Merck Sharp & Dohme, LLC). Additional recommendations for use of PCV20 exist for adults who started their pneumococcal vaccination series with 13-valent PCV (PCV13; Prevnar13; Wyeth Pharmaceuticals, Inc.). The ACIP Pneumococcal Vaccines Work Group employed the Evidence to Recommendations framework to guide its deliberations on PCV21 vaccination among U.S. adults. On June 27, 2024, ACIP recommended a single dose of PCV21 as an option for adults aged ≥19 years for whom PCV is currently recommended. Indications for PCV have not changed from previous recommendations. This report summarizes evidence considered for these recommendations and provides clinical guidance for use of PCV21.

## Introduction

*Streptococcus pneumoniae* (pneumococcus) is a common bacterial cause of respiratory tract infections, bacteremia, and meningitis. Invasive pneumococcal disease (IPD), a pneumococcal infection in a normally sterile site (e.g., blood, cerebrospinal fluid, bone, or joint space), can result in severe morbidity or mortality. Adults with certain underlying conditions or risk factors that increase the risk for pneumococcal disease (risk conditions)* and those aged ≥65 years are at increased risk and have experienced IPD case fatality ratios exceeding 10% (*1*).

The Advisory Committee on Immunization Practices (ACIP) recommends receipt of a pneumococcal conjugate vaccine (PCV) by all adults aged ≥65 years as well as those aged 19–64 years with a risk condition who have not received PCV or whose vaccination history is unknown. Options include either 20-valent PCV (PCV20; Prevnar20; Wyeth Pharmaceuticals, Inc.) alone or 15-valent PCV (PCV15; VAXNEUVANCE; Merck Sharp & Dohme, LLC) followed by 23-valent pneumococcal polysaccharide vaccine (PPSV23; Pneumovax23, Merck Sharp & Dohme, LLC). Additional recommendations for use of PCV20 exist for adults who commenced their pneumococcal vaccination series with 13-valent PCV (PCV13; Prevnar13, Wyeth Pharmaceuticals, Inc.) (*2*).

On June 17, 2024, the Food and Drug Administration licensed 21-valent PCV (PCV21; CAPVAXIVE; Merck Sharp & Dohme, LLC) for use in persons aged ≥18 years (*3*). PCV21 does not contain certain serotypes in other licensed vaccines but adds eight new serotypes (Figure). This report summarizes the evidence considered by ACIP regarding the use of PCV21 for adults, highlighting considerations of immunogenicity, safety, and resource use.

**FIGURE:**
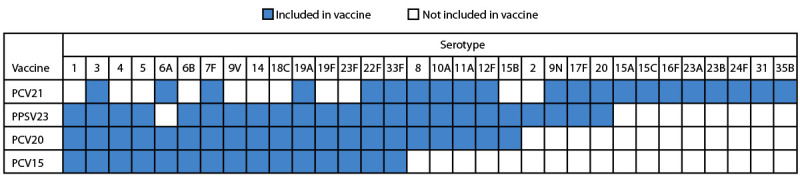
Serotypes*^,^†^^ included in pneumococcal vaccines currently recommended for adults — United States, 2024 **Abbreviations:** PCV = pneumococcal conjugate vaccine; PCV15 = 15-valent PCV; PCV20 = 20-valent PCV; PCV21 = 21-valent PCV; PPSV23 = 23-valent pneumococcal polysaccharide vaccine. * PCV21 is approved for the prevention of invasive pneumococcal disease caused by serotype 15B based upon prespecified criteria for the proportion of participants with fourfold or more rise in OPA responses. https://www.fda.gov/media/179426/download?attachment. ^^† ^^PCV21 contains serotype 20A.

## Methods

During November 2023–June 2024, the ACIP Pneumococcal Vaccines Work Group evaluated the quality of evidence for PCV21 immunogenicity and safety using the Grading of Recommendations, Assessment, Development and Evaluation (GRADE) approach.^†^ Within the Evidence to Recommendations (EtR) framework,^§^ ACIP considered the importance of the public health problem, benefits and harms, values and preferences of the target populations, resource use, equity, acceptability, and feasibility for PCV21 use among adults aged ≥19 years.

## Rationale and Evidence

### Pneumococcal Disease Incidence in Adults Aged ≥19 Years

Before the COVID-19 pandemic, approximately 100,000 noninvasive pneumococcal pneumonia hospitalizations and 30,000 IPD cases occurred annually among U.S. adults (*4*). During 2018–2022, pneumococcal serotypes contained in PCV21 caused approximately 80% of IPD cases among adults with indications for vaccination, including 20%–30% due to the eight new serotypes contained in PCV21 (Supplementary Figure, https://stacks.cdc.gov/view/cdc/160379). Serotype 4, a serotype contained in other licensed pneumococcal vaccines currently in use (PCV15, PCV20, and PPSV23), is not included in PCV21 (Figure). After introduction of the 7-valent PCV in children, serotype 4 IPD significantly declined, but has recently reemerged as a cause of IPD in certain regions, particularly the western United States, namely Alaska (*5*), the Navajo Nation (*6*), Colorado, New Mexico, and Oregon (CDC Active Bacterial Core surveillance, unpublished data, 2022). Affected persons at risk for serotype 4 IPD are typically adults aged <65 years with a risk condition, with history of substance abuse, or who are experiencing homelessness.

Incidence of pneumococcal disease is disproportionately higher in Black or African American (Black) adults than in non-Black adults, resulting in high U.S. societal costs (*4*,*7*,*8*). The introduction of PCV13 among U.S. children reduced disparities that existed in PCV13-type IPD incidence in adults, likely because of indirect effects from PCV13 vaccination in children; remaining racial disparities are primarily due to disease caused by non-PCV13 serotypes^¶^ (*9*).

### PCV21 Immunogenicity

Findings from one phase II (*10*) and three phase III (*11*–*13*) randomized controlled trials (RCTs) compared the immunogenicity of PCV21 (as measured by opsonophagocytic activity** [OPA] geometric mean titers [GMT] and percentage of seroresponders^††^) to that of comparator vaccines (PCV15, PCV20, or PPSV23). One study assessed the safety and immunogenicity of PCV21 with concomitant or sequential administration of the quadrivalent influenza vaccine (QIV) (*13*).

Among immunocompetent, pneumococcal vaccine–naive adults aged ≥50 years, PCV21 met noninferiority criteria for serotypes shared with comparator vaccines (PPSV23 and PCV20) (*10*,*13*). PCV21 elicited statistically significantly higher immune responses for most serotypes unique to PCV21, with the exception of serotype 15C, although the immune response was numerically higher when compared with PCV20 (*13*).

Among immunocompetent adults aged ≥50 years who had previously received a pneumococcal vaccine (PCV13 or PPSV23) ≥1 year before enrollment, PCV21 demonstrated comparable immunogenicity for shared serotypes and was immunogenic for unique serotypes compared with PPSV23 or PCV15; among adults who had previously received PPSV23 followed by or preceded by PCV13, PPSV23 preceded by PCV15, or PCV15 alone, PCV21 was immunogenic for all serotypes (*11*). Among adults aged ≥18 years living with HIV, comparison of recipients of PCV15 followed by PPSV23 8 weeks later with recipients of PCV21 followed by placebo 8 weeks later showed comparable immunogenicity for shared serotypes and was immunogenic for unique serotypes (*12*).

Among immunocompetent adults aged ≥50 years who received PCV21 and QIV concomitantly or sequentially (*14*), coadministration of PCV21 and QIV resulted in numerically lower pneumococcal and influenza antibody titers compared with sequential administration. Coadministration of PCV21 and QIV met noninferiority criteria for immunogenicity^§§^ for all except pneumococcal serotype 23B and influenza strain A/H3N2.

### PCV21 Safety

Safety data from four PCV21 phase III clinical trials (*11*,*13*–*15*) were pooled for the following participants: pneumococcal vaccine–naive adults aged 18–49 years (*13*,*15*), pneumococcal vaccine–naive adults aged ≥50 years (*13*,*14*), and pneumococcal vaccine–experienced adults aged ≥50 years (*11*,*14*). Safety of PCV21 among 4,020 recipients was compared with that among 2,018 recipients of the comparator vaccine (PCV15, PCV20, or PPSV23). The proportion of participants who experienced at least one solicited adverse event was comparable among PCV21 (63.3%) and comparator vaccine (63.9%) recipients. Injection site pain was the most common solicited injection site event (55.6% among all PCV21 versus 54.5% among control vaccine recipients). Among solicited systemic adverse events, the following were more common among all PCV21 recipients than among comparator vaccine recipients: fatigue (27.1% versus 23.7%), headache (18.4% versus 15.5%), and myalgia (11.3% versus 7.5%). Most solicited adverse events were mild (Grade 1) or moderate (Grade 2). Four (0.1%) potentially life-threatening (Grade 4) solicited adverse events were reported (three in PCV21 group and one in the control group). All were fever (≥104.°F [40°C]), which resolved.^¶¶^

Across one phase II clinical trial (*10*) and five phase III clinical trials (*11*–*15*), serious adverse events*** were observed in 74 (1.5%) of 4,963 PCV21 recipients and 49 (2.0%) of 2,472 comparator vaccine recipients through 6 months postvaccination.^†††^ Two serious adverse events, bronchospasm (*14*) and injection site cellulitis (*11*), were deemed to be vaccine-related in the PCV21 recipients; both resolved.

### Economic Analysis

Three economic models (Tulane-CDC, Merck, and Pittsburgh models) (*16*) assessed the cost-effectiveness of using PCV21 in adults who are currently recommended to receive PCV,^§§§^ adults aged 50–64 years, and adults aged 19–49 years. For each model, the primary health outcome used to assess cost-effectiveness was the quality-adjusted life-year (QALY).

Across the three models, base case estimates^¶¶¶^ of using PCV21 instead of PCV20 for adults who are currently recommended to receive a PCV ranged from being cost-saving**** to having a cost of $58,000 per QALY gained. In the Tulane-CDC model, replacing PCV20 with PCV21 in adults who currently have a risk-based vaccine indication resulted in fewer QALYs gained when the proportion of serotype 4 disease among all pneumococcal disease cases was ≥35% (*17*). Base case estimates of using PCV21 in adults aged 50–64 years ranged from $3,000 to $270,000 per QALY gained. Economic models that assessed cost-effectiveness of using PCV21 among adults aged 19–49 years were the least economically favorable among the three groups considered (*16*,*17*).

## Recommendations for Use of PCV21

ACIP recommended PCV21 as an option for adults aged ≥19 years who are currently recommended to receive a dose of PCV. Indications for PCV have not changed since they were previously published (Table) (*2*).

**TABLE:** Clinical guidance for implementing pneumococcal vaccine recommendations for adults aged ≥19 years — United States, 2024

Risk or age group	Vaccine received previously	Options for vaccination
Adults aged ≥65 years	None or PCV7 only at any age	A single dose of PCV21, PCV20, or PCV15. If PCV15 is administered, a single dose of PPSV23* should be administered ≥1 year after the PCV15 dose. A minimum interval of 8 weeks can be considered if PCV15 is used in adults with an immunocompromising condition,^†^ cochlear implant, or CSF leak.
	PPSV23 only	A single dose of PCV21, PCV20, or PCV15 ≥1 year after the last PPSV23 dose.
	PCV13 only	A single dose of PCV21, PCV20, or PPSV23≥1 year after the PCV13 dose. When PPSV23 is used for adults with an immunocompromising condition,^†^ cochlear implant, or CSF leak, administer PPSV23 ≥8 weeks after the PCV13 dose.
	PCV13 at any age and PPSV23 at age <65 years	A single dose of PCV21, PCV20, or PPSV23. If PCV21 or PCV20 is used, it should be administered ≥5 years after the last pneumococcal vaccine dose. If PPSV23 is used, it should be administered ≥1 year after the PCV13 dose (or ≥8 weeks since the PCV13 dose for adults with an immunocompromising condition,^†^ cochlear implant, or CSF leak) and ≥5 years after the previous PPSV23 dose.
	PCV13 at any age and PPSV23 at age ≥65 years	Shared clinical decision-making is recommended regarding administration of either a single dose of PCV21 or PCV20 for any adult aged ≥65 years who has completed the recommended vaccination series with both PCV13 and PPSV23 (i.e., PPSV23 administered at age ≥65 years) but PCV21, PCV20 or PCV15 not yet received. If a decision to administer PCV21 or PCV20 is made, a single dose is recommended ≥5 years after the last pneumococcal vaccine dose.
Adults aged 19–64 years with an immunocompromising condition,^†^ a CSF leak, or a cochlear implant	None or PCV7 only at any age	A single dose of PCV21, PCV20, or PCV15. If PCV15 is used, administer a single dose of PPSV23* ≥8 weeks after the PCV15 dose.
	PPSV23 only	A single dose of PCV21, PCV20, or PCV15 ≥1 year after the last PPSV23 dose.
	PCV13 only	A single dose of PCV21, PCV20, or PPSV23. If PCV21 or PCV20 is used, it should be administered ≥1 year after the PCV13 dose. If PPSV23 is used, administer PPSV23 ≥8 weeks after the PCV13 dose. When PPSV23 is used instead of PCV21 or PCV20 for these adults, a single dose of PCV21, PCV20 or PPSV23 dose is recommended ≥5 years after the first PPSV23 dose.
	PCV13 and 1 dose of PPSV23	A single dose of PCV21 or PCV20, or ≥1 dose of PPSV23. If PCV21 or PCV20 is used, it should be administered ≥5 years after the last pneumococcal vaccine dose. When a second PPSV23 dose is used instead of PCV21 or PCV20, it should be administered ≥8 weeks after the PCV13 dose and ≥5 years after the first PPSV23 dose. The pneumococcal vaccination recommendations should be reviewed again when the person reaches age 65 years. If PCV21 or PCV20 is used in place of any dose of PPSV23, the series is complete, and it need not be followed by additional pneumococcal vaccine doses.
	PCV13 and 2 doses of PPSV23	The pneumococcal vaccination recommendations should be reviewed again when the person turns age 65 years. Alternatively, a single dose of either PCV21 or PCV20 should be administered ≥5 years after the last pneumococcal vaccine dose. If PCV21 or PCV20 is used, the series is complete, and it need not be followed by additional pneumococcal vaccine doses.
Adults aged 19–64 years with chronic medical conditions^§^	None or PCV7 only at any age	A single dose of PCV21, PCV20, or PCV15. If PCV15 is administered, a single dose of PPSV23* should be administered ≥1 year after the PCV15 dose.
	PPSV23 only	A single dose of PCV21, PCV20, or PCV15 ≥1 year after the last PPSV23 dose.
	PCV13 only	A single dose of PCV21, PCV20, or PPSV23 ≥1 year after the PCV13 dose.
	PCV13 and 1 dose of PPSV23	The pneumococcal vaccination recommendations should be reviewed again when the person reaches age 65 years.

**Abbreviations**: CSF = cerebrospinal fluid; PCV = pneumococcal conjugate vaccine; PCV7 = 7-valent PCV; PCV13 = 13-valent PCV; PCV15 = 15-valent PCV; PCV20 = 20-valent PCV; PCV21 = 21-valent PCV; PPSV23 = 23-valent pneumococcal polysaccharide vaccine.

* For adults who have received PCV15 but have not completed their recommended pneumococcal vaccine series with PPSV23, 1 dose of PCV21 or PCV20 may be used if PPSV23 is not available.

^†^ Chronic renal failure, nephrotic syndrome, immunodeficiency, iatrogenic immunosuppression, generalized malignancy, HIV infection, Hodgkin disease, leukemia, lymphoma, multiple myeloma, solid organ transplant, congenital or acquired asplenia, or sickle cell disease or other hemoglobinopathies.

^§^ Alcoholism; chronic heart disease, including congestive heart failure and cardiomyopathies; chronic liver disease; chronic lung disease, including chronic obstructive pulmonary disease, emphysema, and asthma; cigarette smoking; or diabetes mellitus.

### Selection of PCV in Populations with High Proportions of Serotype 4 Pneumococcal Disease

In certain populations in which ≥30% of pneumococcal disease is due to serotype 4, previously recommended pneumococcal vaccines that include serotype 4 (PCV20 alone or PCV15 and PPSV23 in series) are expected to provide broader serotype coverage against locally circulating strains than does PCV21 (Box).

BOX **Clinical guidance on selection of pneumococcal conjugate vaccine in communities with high proportions of serotype 4 pneumococcal disease — United States, 2024**

- PCV21 contains eight pneumococcal serotypes that are not included in previously recommended pneumococcal vaccines (i.e., PCV15, PCV20, and PPSV23). However, PCV21 does not contain certain pneumococcal serotypes that are contained in previously recommended pneumococcal vaccines, one of which is pneumococcal serotype 4.
- In certain adult populations in the western United States, high percentages (i.e., ≥30%) of IPD caused by serotype 4 have occurred. The available IPD serotype data from CDC’s Active Bacterial Core surveillance, as well as similar surveillance from Alaska and the Navajo Nation, indicate that these high percentages are particularly prevalent in Alaska, Colorado, the Navajo Nation, New Mexico, and Oregon. Typically, persons living within these geographic areas who develop serotype 4 IPD are adults aged <65 years with specific underlying conditions or risk factors, such as alcoholism, chronic lung disease, cigarette smoking, homelessness, and injection drug use. Importantly, these persons usually have not received a pneumococcal conjugate vaccine containing serotype 4. In such populations, other recommended pneumococcal vaccines (e.g., PCV20 alone or both PCV15 and PPSV23) are expected to provide broader serotype coverage against locally circulating strains compared with PCV21.
- The percentages of serotype 4 IPD cases in other areas of the western United States without IPD surveillance are currently unknown. IPD surveillance from other geographic areas in the United States (e.g., midwestern, eastern, and southern regions) has not detected significant percentages of serotype 4.
- This clinical guidance will be reviewed and updated as pneumococcal disease epidemiology evolves.

**Abbreviations:** IPD = invasive pneumococcal disease; PCV = pneumococcal conjugate vaccine; PCV7 = 7-valent PCV; PCV13 = 13-valent PCV; PCV15 = 15-valent PCV; PCV20 = 20-valent PCV; PCV21 = 21-valent PCV; PPSV23 = 23-valent pneumococcal polysaccharide vaccine.

### Coadministration with Other Vaccines

In accordance with ACIP’s General Best Practice Guidelines for Immunization, routine administration of a pneumococcal vaccine with other age-appropriate doses of vaccines at the same visit is recommended for adults who have no specific contraindications to vaccination at the time of the health care visit (*18*).

### Contraindications and Precautions

Vaccination providers should consult the vaccine package insert for precautions, warnings, and contraindications (*19*). Vaccination with PCV or PPSV23 is contraindicated in persons known to have had a severe allergic reaction (e.g., anaphylaxis) to any component of the vaccine. PCVs are also contraindicated in persons known to have had a severe allergic reaction to any diphtheria toxoid–containing vaccine (*2*,*19*).

## Reporting of Vaccine Adverse Events

Adverse events occurring after administration of any vaccine should be reported to the Vaccine Adverse Event Reporting System (VAERS). Instructions for reporting to VAERS are available at https://vaers.hhs.gov/reportevent.html or by calling 800-822-7967. 

## Future Research and Monitoring Priorities

CDC and ACIP will assess available data to evaluate whether evidence supports lowering the age for the current adult age-based pneumococcal vaccination recommendation. CDC and ACIP will continue to assess safety and public health impact of PCVs (i.e., PCV15, PCV20, and PCV21) among adults.

## Footnotes

* Alcoholism; cerebrospinal fluid leak; chronic heart, liver, or lung disease; chronic renal failure; cigarette smoking; cochlear implant; congenital or acquired asplenia; diabetes mellitus; generalized malignancy; HIV; Hodgkin disease; immunodeficiency; iatrogenic immunosuppression; leukemia, lymphoma, or multiple myeloma; nephrotic syndrome; solid organ transplant; or sickle cell disease or other hemoglobinopathies.

† https://www.cdc.gov/vaccines/acip/recs/grade/PCV21-non-risk-based-adults-19-49.html; https://www.cdc.gov/vaccines/acip/recs/grade/PCV21-non-risk-based-adults-50-64.html; https://www.cdc.gov/vaccines/acip/recs/grade/PCV21-adults-19-and-older.html

§ https://www.cdc.gov/vaccines/acip/recs/grade/PCV21-non-risk-based-adults-19-49-etr.html; https://www.cdc.gov/vaccines/acip/recs/grade/PCV21-non-risk-based-adults-50-64-etr.html; https://www.cdc.gov/vaccines/acip/recs/grade/PCV21-adults-19-and-older-etr.html

¶ Pneumococcal serotypes 22F, 33F, 8, 10A, 11A, 12F, 9N, 17F, 20, 15A, 15C, 16F, 23A, 23B, 24F, 31 and 35B are non-PCV13 serotypes contained in PCV21.

** Opsonophagocytosis refers to engulfment of bacteria that have been coated with immunoglobulin G antibodies by phagocytic cells (e.g., macrophages or neutrophils).

†† Defined as proportion of participants with a fourfold or more rise in serotype-specific OPA responses.

§§ Defined as two-sided 95% CI of the pneumococcal serotype-specific OPA GMT ratio (concomitant group/sequential group) to be >0.50 and influenza strain-specific hemagglutination assay GMT ratio (concomitant group/sequential group) to be >0.67.

¶¶ No occurrences of Guillain-Barré syndrome were observed.

*** Defined as any untoward medical consequence that results in death, is life-threatening, requires inpatient hospitalization or prolongs existing hospitalization, results in persistent or significant disability or incapacity, is a congenital anomaly or birth defect, or is another important medical event.

††† PCV21 recipient group includes participants who received PCV21 and concomitant influenza vaccine, and participants who received influenza vaccine followed by PCV21.

§§§ Adults aged 19–64 years with risk-based vaccine indication, and all adults aged ≥65 years were considered in the economic models.

¶¶¶ A base case estimate in cost-effectiveness analysis is the primary scenario used to assess an intervention's costs and health outcomes. It serves as a benchmark for comparison with alternative scenarios and sensitivity analyses to guide health policy decisions.

**** Lower cost and improved health outcomes compared with the comparator strategy.
